# DIRECT OBSERVATION OF OLDER ADULTS VIDEO TELEMEDICINE CHALLENGES

**DOI:** 10.1093/geroni/igad104.0475

**Published:** 2023-12-21

**Authors:** Lauren Moo, Maria Venegas, Chelsea Hawley, Megan McCullough, William Hung

**Affiliations:** Veterans Health Administration, Bedford, Massachusetts, United States; Veteran Affairs, Bedford, Massachusetts, United States; VA Bedford Healthcare System, Bedford, Massachusetts, United States; VA Bedford Healthcare System, Bedford, Massachusetts, United States; Veterans Health Administration, New York City, New York, United States

## Abstract

Video to home has the potential to increase access to clinical services for older adults that face barriers to in-person care and to specialty care such as mobility and transportation challenges. There is evidence that the digital divide has been increasing rather than shrinking for this population. Twenty older cognitively intact Veterans enrolled in Aim 1 of a study of video visits with clinical pharmacists and allowed the study team to observe them in their homes as they prepared for and attempted to connect for the virtual encounter. Technology challenges were encountered in more than 80%, including 60% that required assistance from the in-person study staff, despite these being highly motivated participants. We will share both quantitative and qualitative data regarding the nature of the challenges observed and discuss the implications for geriatric tech equity.

